# Negotiating Medicare Drug Prices: A New Attempt to Control Purchase Prices

**DOI:** 10.1017/jme.2025.59

**Published:** 2025

**Authors:** Marc A. Rodwin

**Affiliations:** School of Law, Suffolk University, Boston, United States

**Keywords:** Inflation Reduction Act, Negotiation, Pharmaceutical, Prices, Health Technology Assessment

## Abstract

The Inflation Reduction Act (IRA) creates a new process to cap Medicare Part D branded drug prices. It prohibits Medicare from paying more than a specified discount from average private market prices and requires that CMS negotiate with manufacturers to agree on a maximum fair price that Medicare will pay that is lower than the specified discount. This article analyzes the cause of high drug prices and how negotiations to set the maximum fair price might unfold. It compares Medicare’s new pricing process to the way drug prices are set in Medicaid, the Veterans Administration, U.S. private insurers, and European nations. It analyzes how negotiations to set the maximum fair price might unfold in light of negotiation theory and the practices to negotiate prices employed in Europe. It draws inferences from the initial published data on the first round of negotiated prices.

This article analyzes recent federal legislation, the Inflation Reduction Act (IRA). The legislation aims to reduce Medicare spending on branded prescription drugs purchased and used outside of hospitals by requiring that Medicare pay no more than a specified discount from average market prices and, in addition, by creating a process for Medicare to negotiate prices below that discount rate. High purchase prices for branded, patent-protected drugs, rather than the cost of generic drugs, drives the US’s high pharmaceutical spending.

We begin with a review of data on US drug prices in comparison to other nations. To help assess policies to control prices, we analyze the causes of high drug prices. Since US federal programs sometimes mandate a specified discount of private market prices, we examine the way US private insurers set prices. In addition, we discuss current payment policies for Medicaid and the Veterans Administration (VA), which historically have paid lower prices for drugs than Medicare. The remainder of the article explores the IRA reforms. We analyze the process used to determine the minimum discount Medicare will receive, the process employed to negotiate drug prices, how those negotiations might play out, and the effect of the Medicare negotiated prices on prices outside of Medicare. We also contrast Medicare’s drug price negotiation to price negotiation and cost control under private US insurance and in selected European countries.

US prices for branded prescription drugs are significantly higher than other OECD nations. A study of 2022 data for international drug prices by Rand Corporation found that branded drugs were more than four times as high in the US as in other nations.^1^ Furthermore, US prices vary widely, because the US lacks a uniform prescription drug pricing policy. Coverage, purchase prices, patient cost-sharing, choice of medicines, and drug access vary across multiple private and public insurance programs. In 2022, Americans insurance coverage was apportioned as follows: private employer-sponsored insurance (48.7%); private individual: (6.3%); Medicaid (21.2%); Medicare (14.6%); and armed services insurance (1.3%). Eight percent were uninsured.^2^

Each private insurer is responsible for negotiating the prices it pays to purchase branded pharmaceuticals and to determine the copayment that patients pay for medications. Typically, they delegate these decisions to pharmacy benefit managers (PBMs) with which they contract. Similarly, Medicare, Medicaid, the Veterans Administration (and other federal programs, such as the Indian Health Service, Department of Defense, and Public Health Service) each have distinct policies that influence their purchase prices and patient copayments.

A related but distinct issue is the high patient copayments for prescription drugs. A typical private health plan offered by employers has four tiers of drugs with different patient copayments or coinsurance for drugs in each tier. In 2022, the average payment for up to a 30-day supply of drugs in the first tier (generic drugs) required an $11 copayment. The second tier for low-cost branded drugs required a $36 copayment; drugs in the third tier required on average a $66 copayment; and the fourth tier, for specialty drugs, required a $125 copayment.^3^ Actual copayments vary with the drug and health insurance plan.

## The Main Causes of High Drug Prices: Monopolies and Insurance

Drug development is costly. It requires drug discovery, clinical trials to test drugs for safety and effectiveness, and purchase of intellectual property. However, the federal government typically subsidizes a substantial share of research and development (R&D), and high R&D costs only partly explain high prices. Two characteristics of the pharmaceutical market drive high prices. First, patents and US Food and Drug Administration (FDA) grants of market exclusivity preclude market competition that might otherwise restrain prices. Second, health insurance removes the usual budget cap — the individual patient’s disposable income — that sets a ceiling on what pharmaceutical firms can charge.

US law grants patents to inventors to provide incentives for R&D. Pharmaceutical patents create an exclusive right to sell a drug for a limited duration, typically 20 years, in return for disclosure of the invention so that others can use it after the patent’s expiration. During the patent, the patent holder lacks competitors that can market the same invention. By definition, patents create a monopoly, which removes price restraints due to competition. Of course, patents only preclude competition from selling the patented invention. The patented drug might have competitors from alternative drugs or non-drug therapies that treat the same illness. Nevertheless, patents significantly reduce the most direct competition.

The anticompetitive effects of patents are amplified by market exclusivity granted by the FDA and other national drug registration authorities. Market exclusivity can extend the duration of protection from competition and sometimes precludes competition even from products that compete without infringing the original drug’s patent. The FDA grants market exclusivity for drugs that are new chemical entities (5 years), to compensate for the time it takes the FDA to review new drug applications (up to 2 years), for orphan drugs (7 years), for biologics (12 years), for certain antibiotics (7 years), and for conducting a study of an approved drug’s use in pediatric populations (6 months).

Even when manufacturers lack competition due to patents, their prices are still limited by the consumer’s ability to pay, which is usually capped by their disposable income. Typically, when manufacturers raise their prices, their sales volume decreases. As a result, manufacturers often restrain their prices because increased sales volume with lower prices makes up for the lower per unit price. The market for pharmaceuticals and medical care, however, differs because insurance pays most of the cost. Insured individuals with low or average income, therefore, can purchase medicines sold at much higher prices than they could otherwise pay, and manufacturers can set prices without considering an individual’s income as a constraint.

Take, for example, the pricing of anti-cancer drugs, where recently approved therapies have been priced in excess of $283,000 annually.^4^ Since median household income in the US in 2023 was $80,610,^5^ many people lack the income to purchase such drugs if they pay the full cost out of pocket. If pharmaceutical companies had to sell drugs to individuals without insurance they would have to lower the price or lose most of their market. However, with insurance companies footing the cost, manufacturers can charge the current high prices unless some other factors restrict what manufacturers can demand.

### Countering the Inflationary Effects of Monopolies and Insurance on Prices in Europe

Addressing high pharmaceutical prices requires controlling the power of monopolies and the inflationary effects of insurance. European nations have had an easier time than the US controlling pharmaceutical spending because they often have a national health insurance system that is the sole or dominant purchaser. The market is therefore a bilateral monopoly with the insurer and manufacturer negotiating a price based on their respective bargaining power. When European nations, such as Germany, have multiple health insurers, public policies coordinate their purchasing power so that regulations set a maximum purchase price for all insurers, so the effect is similar to that of a single insurer.

European nations employ health technology assessment (HTA) to cap the purchase price of pharmaceuticals. For example, the United Kingdom and the Nordic countries rely mainly on cost-benefit analysis to determine the value of new drugs and pay no more than is cost-effective.^6^ Cost-effectiveness analysis is well known in the US. For example, the Institute for Clinical and Economic Review (ICER), an independent non-profit organization, conducts a cost-effectiveness analysis of health technologies for public or private sector entities that employ it, but the federal and state governments do not grant ICER any official status or require that purchasers employ its analysis or cap prices based on its cost-effectiveness analysis. Although some private payers require a determination of a medicine’s value for money before it is put on a low-tiered formulary, the federal government does not require the use of HTA to cap prices in the public or private sector.

In a similar vein, France, Germany and other continental nations employ a different sort of HTA. They compare the effectiveness of a new drug to older comparable therapies and pay no more than the older therapy unless the new drug provides added therapeutic benefit.^7^ New drugs without added therapeutic benefit are assigned to a reference price group, all of which receive no more than a common maximum purchase price, although they can compete for market share by lowering their prices. Generally, once European nations establish a maximum sale price they do not allow price increases, even to correct for inflation.

In contrast, the US has neither a national health insurance system that is a single or dominant purchaser, nor a system that requires HTA to cap pharmaceutical purchase prices.

## Pharmaceutical Pricing Under US Private Insurance

Most Americans have health insurance through a private insurer or employer self-funded insurance plan, which are subject to overlapping state and federal regulation. Regarding coverage for drugs, state governments sometimes have mandates for certain drug coverage. For example, many states require insurers to cover anti-cancer drugs even for uses not approved by the FDA.^8^ However, typically it is the insurers or their designated PBMs who choose which particular products to include in their formulary.

Private insurers usually delegate purchasing of pharmaceuticals, and administration of their drug formulary, to PBMs. ^9^ PBMs negotiate rebates and other price discounts from drug manufacturers’ list prices, or from some private market benchmark price, such as average wholesale price or wholesale acquisition cost, which are public.^10^ However, net prices — namely the amount individual insurers pay after all rebates and other discounts — remain confidential.

The discounts received vary based on the volume purchased, purchaser leverage, whether there are competing therapies. and the burden of the illness. In addition, typically, unless the manufacturer offers a sufficient rebate, PBMs restrict patient access to expensive branded medicines. For example, PBMs often place high-priced drugs in a formulary tier that requires substantial patient co-payments. A 2023 survey of employer-sponsored health plans found that co-payment rates were on average 20% to 28% of the cost and ranged from $11 to $125 per prescription, depending on the tier the drug was assigned.^11^ Consequently, many patients either choose a lower-cost medication or forgo the use of the drug.^12^

PBMs also often require physicians and patients to obtain insurer approval before authorizing coverage for high-cost drugs, which insurers grant only if the doctor convinces the insurer that the medication is medically necessary and that no other appropriate therapeutic alternative exists. Still, other PBM policies may require a patient to fail on a therapeutic alternative before authorizing the use of the higher-cost drug. These policies reduce the manufacturers’ sales. PBMs leverage removal or reduction of these restrictions in return for an acceptable discount from the manufacturer. In a similar vein, where a PBM has a choice of medicines from various manufacturers to include in a formulary, they may negotiate an exclusive or privileged contract with one drug manufacturer in return for price discounts.

PBMs argue that they are a means to control pharmaceutical spending; however, it is not clear the extent to which they do so or whether they contribute to high pharmaceutical spending. Many critics of PBMs have proposed to reform their operation.^13^ While PBMs negotiate discounts from list prices, compensation to PBMs is often a percentage of any reduction from list price, which reduces the value of the discount and also provides incentives for manufacturers to raise list prices. However, PBM compensation varies and in recent years there has been a move to return a greater share of rebates to insurers.^14^

Indeed, manufacturers often argue that the driver behind rising drug prices is the PBM compensation model, rather than increasing manufacturer net gain.^15^ In addition, while discounts from the average market price help the insurer who receives the discount, it does not affect high average market price, and manufacturers can set their list prices anticipating their need to offer discounts. Furthermore, PBMs sometimes earn income through fees paid by insurers, patients, and pharmaceutical firms, generating significant conflicts of interest.^16^ A *New York Times* investigation found PBMs often favor selection of drugs where they earn higher income even if the cost to purchaser and/or patient co-payments are higher than for alternatives.^17^ Finally, PBMs’ cost control measures have a cost for patient care: restrictions on patient access and caregiver choices that are often not in the interest of patients.

### Pharmaceutical Pricing in US Government Health Insurance Programs

The US has several government-funded health insurance programs, each controlled by a unique pharmaceutical pricing policy.

### Medicaid

Medicaid provides insurance for select low-income populations. It is jointly administered and funded by the federal government, specifically the Centers for Medicare and Medicaid Services (CMS), and each participating state.

Most drug manufacturers participate in a Medicaid pricing discount program. In return for paying no more than the best net US price or a 23.1% discount from the average private market price, whichever yields a lower price, Medicaid includes all of the manufacturer’s products in its formulary, even when there are less expensive alternatives.^18^ All state-administered Medicaid programs can purchase drugs at these prices. Additionally, all but three state Medicaid programs have secured additional manufacturer rebates in return for removal or reduction in restrictions, such as prior authorization, used to limit prescription drug use. And finally, since 1990, manufacturers cannot raise the prices Medicaid pays for prescription drugs greater than the annual rate of inflation.^19^ Nevertheless, because each state Medicaid program negotiates the prices they pay for prescription drugs separately, the bargaining power of state programs is limited.

### The Veterans Administration

The Veterans Health Administration (VA) provides healthcare coverage to US armed services veterans, primarily providing healthcare and services through its own facilities. The VA is not subject to state law regarding insurance regulation and coverage mandates. Under federal law, the VA will pay no more for drugs than the federal Medicaid price, and it has the right to negotiate its own prices. As such, the VA often negotiates prices even lower than Medicaid.^20^ The VA’s bargaining leverage is derived from its control over its drug formulary and prescriptions. The VA can decline to include in its formulary drugs deemed too expensive and can limit alternatives when more than one drug is available for a therapeutic purpose. Such policies guide prescribing practices and can restrict drug sales, which in turn provide leverage to secure discount prices.

### Medicare Drug Prices Under the Medicare Modernization Act of 1983

Enacted in 1965, Medicare covers individuals over 65 years, as well as, regardless of age, people with permanent disabilities and two diseases: end stage renal disease and Amyotrophic Lateral Sclerosis (ALS). Administered and funded by the Center for Medical Services (CMS), Medicare did not initially provide drugs for use outside of hospitals. However, more recently, patients who received Medicare benefits through private insurers under Medicare Part C sometimes received outpatient prescription drug coverage. Adding a prescription drug benefit for all beneficiaries was a goal proposed by Democratic party legislators for many years, but there was insufficient Congressional support to enact legislation for a drug benefit due to Republican party opposition until 2003.

Enacted at the behest of President George W. Bush (Republican), the Medicare Modernization Act (MMA) established Medicare Part D. Since the MMA came into effect in 2006, Medicare subsidizes federally regulated insurance offered through competing private insurers offering outpatient drug coverage. Each insurer independently sets their premium.^21^

When enacting the MMA, the Bush Administration and Congress opted to rely on private insurers to negotiate the pharmaceutical purchase prices.^22^ In fact, the legislation prohibits CMS from what it refers to as *interference*, namely any direct involvement in drug pricing. The MMA’s prohibition on Medicare’s interference on pricing differs from Medicare policy for other services. Medicare has created specialized payment systems to set prices for hospitals, physicians, and other services. For example, federal legislation established a Medicare payment system under which hospitals are paid using a prospective payment based on the patient’s primary diagnosis. Other federal legislation sets up a Medicare payment system for physician fees that uses a resource-based relative value scale for approximately 10,000 services, each of which is referenced by a separate billing code. These pricing policies have been accepted by hospitals and physicians, and even adopted as the payment method used by private insurers.

Between 1990 and 2000, the Democratic party proposed legislation seeking to add an outpatient drug benefit to Medicare using a framework controlled by the federal government, but no bill was enacted. However, following the election of George W. Bush in 2000, the White House and Republican-controlled Congress promoted a different approach, whereby insurers and pharmaceutical firms determined prices independent of CMS regulation or oversight.^23^ Most Democratic members of Congress opposed the Bush administration proposal because it promoted a private insurance model and restriction on government setting or negotiating prices. A few Democrats, however, voted for the legislation, believing that this was a necessary compromise to ensure that all Medicare beneficiaries willing to pay a premium could receive drug coverage.

The MMA also required that private insurers offering the outpatient drug benefit cover all drugs in six therapeutic classes (immune suppressants, antidepressants, antipsychotics, anticonvulsants, antiretrovirals, and antineoplastics) and at least two drugs in all other therapeutic classes.^24^ As such, Medicare drug plans had to include certain prescription drugs, regardless of their price. As a consequence, Medicare drug plans pay higher prices than other federal programs and typically more than other private insurers — and served as the impetus for proposals to reform Medicare outpatient drug pricing.^25^

## Legislative Proposals to Control Drug Prices Following the Medicare Modernization Act

Despite public concerns regarding the high prices of Medicare drugs, support for the MMA’s prohibition on CMS’s involvement in drug pricing remained strong between 2000 and 2020, with Republican party legislators blocking any legislation that eliminated the ban. During this time, however, two principal strategies to control Medicare’s drug costs were proposed by Congress or the President.^26^

The first strategy would cap Medicare drug prices by reference to European or international drug prices. Often referred to as external reference pricing, this strategy emulated policies implemented by 25 European nations.^27^ Using this strategy, the price cap for a drug in one country is set by referencing the maximum official price for the drug in another country or a group of countries. Similarly, the lowest price might be determined by referencing the lowest price paid in another country.^28^ For example, in France, for drugs with added therapeutic benefit, public authorities look to prices in Germany, the UK, Italy, and Spain and pay no more than the highest price of these nations and no less than the lowest price paid by these nations. In Europe the reference price does not set the final price but provides a framework for negotiating a price. Under the US proposals, however, the Medicare price would be calculated based on an index of the prices of the selected countries.^29^

Following this European model, some of the legislation proposed in Congress capped Medicare drug prices at the European or international reference price for the drug.^30^ Other legislative proposals during this time capped Medicare prices at 120% of the European reference price.^31^ Specifically, the first Trump administration initially proposed regulations under which Medicare would pay no more than an international reference price based on the average price paid by 16 developed nations.^32^ Later, the Trump administration proposed an alternative policy requiring Medicare to pay no more than the best price of any similarly situated country.^33^ However, the reference prices employed in all of these proposals were the *official* maximum price; virtually all European nations receive confidential discounts from the official price.^34^ Therefore, Medicare would have continued to pay more than the net price paid by the reference price countries under these proposals.

A second strategy was similar to that employed by Medicaid: manufacturers would have to grant Medicare a specified discount from the average non-federal US private market price. However, without any policy to control private market launch list prices, or annual increases, manufacturers could easily increase their revenue by setting launch prices that anticipated the discounts required by legislation. Manufacturers can raise prices because they lack significant market competition on their patented drug and are not subject to significant budget constraints due to insurance coverage. Consequently, this pricing strategy is likely to yield limited reductions in federal or national outpatient drug spending compared to the use of HTA or the other strategies employed by European nations.

The US had an office of technology assessment in the 1970s that evaluated technologies, but Congress defunded the agency in 1995, in part due to lobbying from entities that objected to their technologies being evaluated.^35^ The US has also had other entities that evaluated selected technologies.^36^ Despite or perhaps because of this history, I have found no bills introduced in Congress that would incorporate HTA as a method by which to cap Medicare prices despite HTA being used to set pharmaceutical prices in most European nations.^37^ This absence is ironic because policymakers were aware that European nations paid lower prices and proposed reforms that would cap Medicare prices at prices that European nations paid. However, they could have achieved these results more effectively and controlled the process if they had instead adopted a similar form of HTA to cap Medicare prices.

## The Inflation Reduction Act of 2022 Introduces Price Caps and Negotiated Drug Pricing

### Key Elements of the IRA

Following a shift from Republican to Democratic control in both the presidency and the Senate in 2020, Congress passed the Inflation Reduction Act (IRA) in August 2022 by a majority and one vote. The IRA requires manufactures to pay rebates on drugs if prices rise faster than inflation for drug reimbursed under Medicare Part B and outpatient drugs under Medicare part D.^38^ It also revised the 340B drug pricing program aimed to support hospitals disproportionally caring for underserved populations. Most notably, the IRA carved out an exception to the MMA prohibition on negotiating Medicare Part D prices for some drugs, and caps annual copayments to $2,000 in 2025, caps the cost of insulin at $35 a month and covers recommended adult vaccines without cost sharing.^39^

The IRA changes Medicare Part D drug spending in two main ways. First, the IRA caps the annual price increases of branded prescription drugs paid by Medicare at the level of inflation. As half of Medicare Part D covered drugs experienced annual price increases higher than inflation from 2018 to 2020, this change is significant.^40^ Second, and even more important, the IRA carves out an exemption to the MMA prohibition on federal government involvement on capping or negotiating prices paid by Medicare.^41^ In effect, this transforms CMS from being a price taker to being something close to a price setter for the drugs with negotiated prices. The law allows CMS, starting in 2024, to negotiate drug prices directly with manufacturers. The negotiated prices, announced in August 2024, for the first 10 selected single-source branded drugs will take effect in 2026. Fifteen additional negotiated prices will go into effect in 2027 and 2028, with 20 more added in 2029. The initial bill allowed private insurers to purchase the drugs at the same price as Medicare if they could not purchase the drugs for less on their own. However, the Congressional parliamentarian ruled that because the legislation was initiated as a budget reconciliation measure authorized only for policies directly affecting government spending, it could not include the provision extending the negotiated prices to private insurers outside of the Medicare program.

Certain features curb the IRA’s impact. Most notably, the legislation only applies to drugs marketed for a designated number of years: a small molecule drug must be on the market for nine years, while a biologic must be on the market for 13 years before eligible for negotiation. Considering the average length of market exclusivity, commonly 12 and 16 years for small molecule drugs and biologics, respectively, the IRA drug pricing rule does not affect prices for more than half of the drug’s patent life.^42^ At the end of market exclusivity, drugs typically encounter price competition from generics and manufacturers either lower prices or lose the overwhelming share of the market, independent of CMS’s power to negotiate.

### The IRA’s Pricing Formula

**Figure 1.**
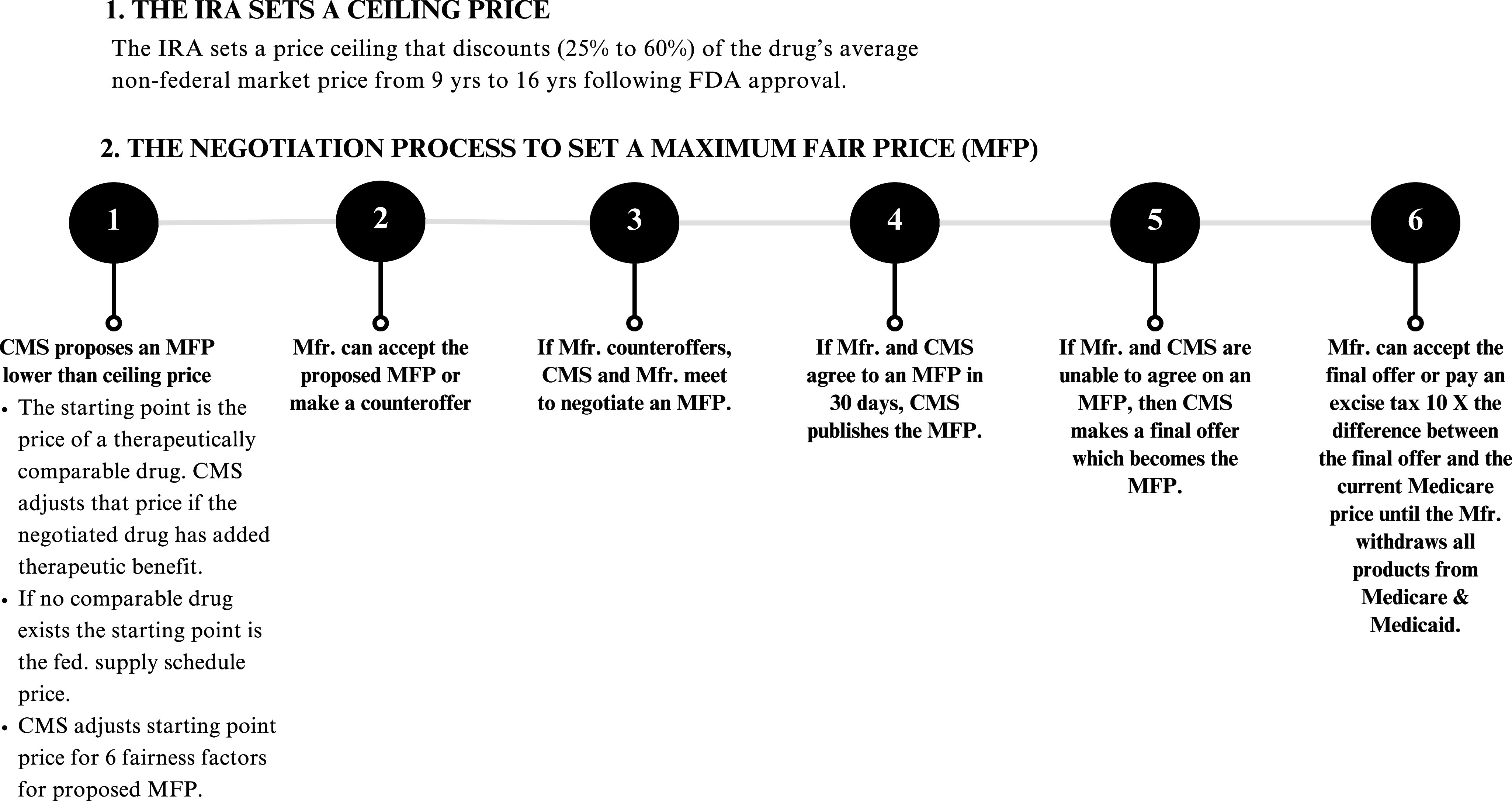
The Process to Determine the Maximum Fair Price.

For the selected drugs, the IRA mandates a minimum discount from average private market price (referred to as the ceiling price) and also sets up a process for CMS and the manufacturer to negotiate a maximum fair price (MFP) (see Figure 1). There are no such regulations that oversee how the VA negotiates its prices. The aim of the IRA negotiation rules is that the MFP should be lower than the statutory ceiling price if possible. Medicare and the Part D insurance plans will not be allowed to pay a price higher than the MFP. The IRA dictates specific parameters for the MFP.

First, the MFP cannot be any higher than the price that Medicare currently pays. The technical provision for determining Medicare’s current price is either (1) the drug’s enrollment-weighted Part D current net price after accounting for all rebates and price concessions or (2) the average sales price paid by all non-federal government drug purchasers after rebates and discounts.

Second, the IRA caps the MFP at a specified discount from the current average private market price. The discounted price, as mentioned above, is referred to as the ceiling price. For small molecule drugs, the ceiling price is a 25% discount from the US average private market price for drugs marketed between 9 and 12 years. The discount rate increases to 35% for drugs marketed between 12 and 16 years, and to 60% for drugs marketed for 16 years or more.

Third, the IRA sets up a process for CMS and the manufacturer to negotiate the MFP that is no higher than the statutorily set ceiling price. CMS must develop and employ a consistent methodology to generate a price that it proposes to the manufacturer, and then negotiate with the manufacturer to attempt to reach an agreement on the MFP. CMS developed a basic methodology characterized by two key steps.^43^

First, CMS identifies the Medicare price of a therapeutically comparable drug and adjusts this price for any additional benefit of the drug whose price is being negotiated. This price is referred to as the starting point price. In cases where no comparable drug is available, CMS takes as the starting point price the Federal Supply Schedule price, that is, the lowest available price available to the VA, Department of Defense, Public Health Service, and Coast Guard.

### Price Adjustments for Fairness

**Figure 2.**
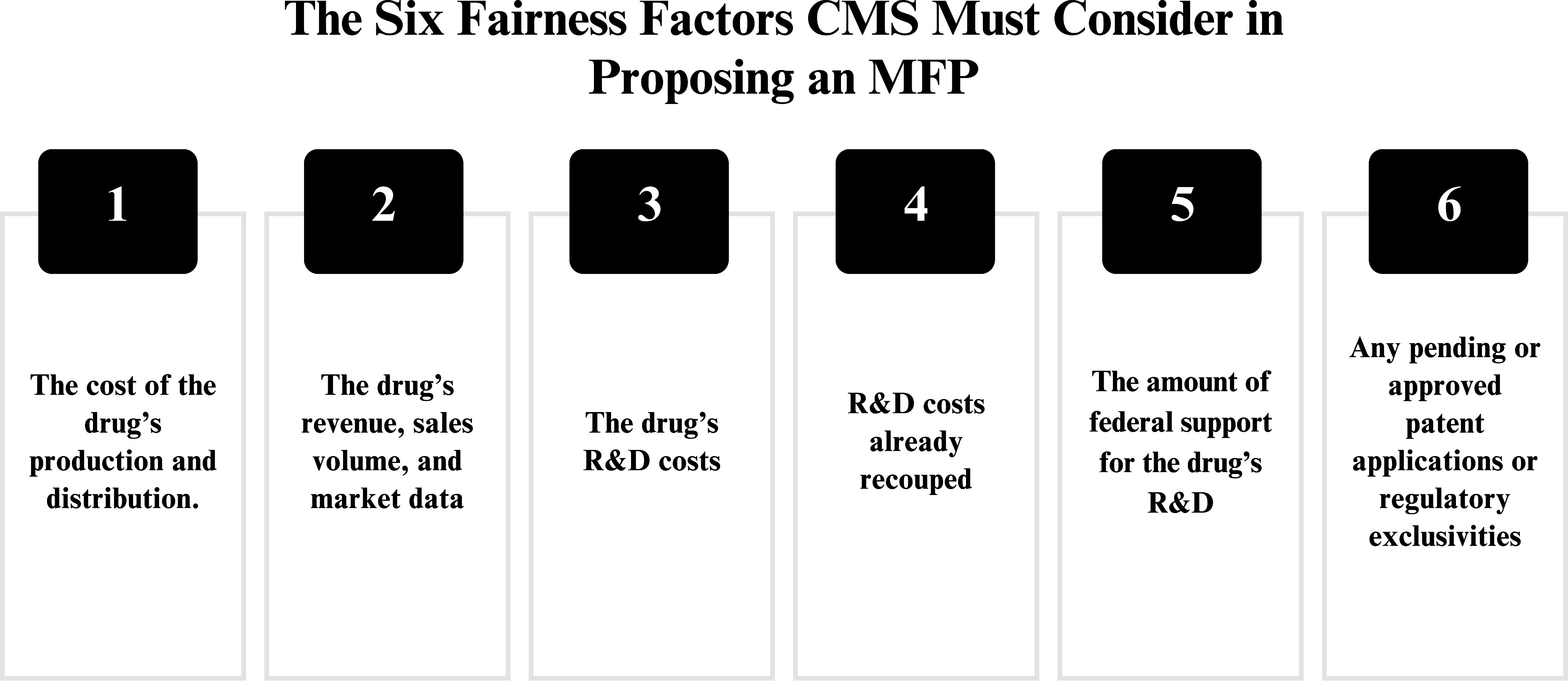
The Siex Fiarness Factors CMS Must Consider in Proposing an MFP.

CMS next adjusts the starting point price for “fairness” by taking account of six factors. The IRA requires that when formulating its proposed MFP, CMS consider six fairness factors and that CMS refer to these factors to justify its proposal (see Figure 2). The manufacturer also must refer to these six factors to support any counteroffer.

The IRA does not specify an algorithm or explain how CMS should weigh fairness factors to modify the starting point price, so CMS has considerable discretion. In short, the legislative requirement to consider six fairness factors does not set an upper or lower limit on CMS’s proposed price or the manufacturers’ counteroffer. Furthermore, the IRA does not mention other factors that bear on fairness, such as the price paid for the products by Medicaid, the VA or purchasers in other nations. Nor does it refer to the manufacturer’s rate of return, i.e., its profit, which is usually considered important to determining a fair price.

Let us consider in more detail each of the six fairness factors. The first factor, cost of production and distribution, suggests that the MFP should compensate at least for those costs, but does not indicate how much over the R&D costs constitutes a minimum or maximum fair return. The second factor, the drug’s revenue, sales volume and other market data, provides information needed to calculate the effect of a price on the manufacturer’s revenue and its relation to the manufacturer’s cost of production and distribution. The sixth factor, pending and approved patient applications and regulatory exclusivities, helps estimate the manufacturer’s ability to restrict competition in the future, and thereby counts on continued high sales revenue.

The third and fourth factors (the drug’s R&D costs and the R&D costs already recouped) suggest that R&D costs should be discounted as a fairness factor after they are recouped. But how should R&D costs be accounted for? By the time the negotiations start, the manufacturer’s previous revenue will almost certainly have exceeded R&D costs. Should R&D costs be amortized over the life of the drug rather than paid back as soon as earnings exceed those costs? What assumptions should CMS make about a fair annual rate of return until the R&D costs are recouped? The fifth factor, the amount of federal support for R&D, suggests that CMS should reduce total R&D costs by the amount of federal support. However, it is unclear whether federal R&D subsidies should reduce what CMS considers to be a fair rate of return. The sixth factor, pending and approved patent applications or regulatory exclusivities, could justify a lower MFP since the manufacturer will gain monopoly pricing from the new market exclusivities.

### The IRA’s Price Negotiation Process

After CMS calculates a proposed MFP and submits it to the manufacturer, CMS and the manufacturer negotiate. The manufacturer has 30 days to accept CMS’s offer or make a counteroffer, which must be supported by manufacturer-supplied data related to the six fairness factors. CMS and the manufacturer can meet to discuss any counteroffer and negotiate a final MFP, which CMS then publishes in the Federal Register. All negotiation discussions remain confidential unless the manufacturer discloses information, in which case CMS can disclose information in response.

If CMS and the manufacturer fail to agree on an MFP within 30 days, CMS makes a final offer which the manufacturer may accept or reject before CMS publishes its final offer as the MFP. If the manufacturer rejects CMS’s final offer, the IRA requires the manufacturer to pay an excise tax 10 times the difference between the final offer and any current manufacturer sale price to Medicare. The manufacturer’s tax liability will end only by its acceptance of the final offer or by terminating its sales of the negotiated drug and all its other products to Medicare and Medicaid.

### How Negotiation Under the IRA Might Play Out

CMS and the manufacturer will not easily agree on an MFP. Each party will apply the six fairness factors based on their own perspective. They will also make different inferences regarding what price would be fair for Medicare to pay when they look to the prices paid by other purchasers of the drug, including Medicaid, the VA, and European nations. Given that each party will pursue its own interests, how might they reach an agreement?^44^

In any negotiation, all parties seek the outcome most favorable to their interests and concessions are generally driven by each side’s perception of their *Best Alternative to a Negotiated Agreement* (BATNA), that is, the default outcome in the absence of an agreement.^45^ Unless they make a mistake, neither party will accept an agreement less favorable than their BATNA. Typically, there is a *Zone of Possible Agreement* (the ZOPA) which includes outcomes superior to both parties’ BATNA, yet each side would like the other to make concessions. The party with the most to lose from the absence of an agreement has the least amount of leverage and will usually make the most concessions to reach an agreement, while the party with the least to lose has the most leverage and typically make few concessions.^46^

In principle, CMS is in a stronger position than the manufacturer in negotiation because without an agreement, CMS’s final offer becomes the MFP. However, publishing an MFP without an agreement entails risk for CMS. If the manufacturer rejects CMS’s final offer and terminates all sales to Medicare and Medicaid, that would restrict patient access to medications and likely create a political crisis.

Further weakening CMS’s bargaining leverage is the risk that without an acceptable agreement, manufacturers would lobby for, and Congress would enact, legislation that weakens or repeals the IRA. The industry is already litigating to overturn the IRA drug pricing provisions.^47^ For 20 years, CMS was precluded from negotiating pharmaceutical prices, largely due to the strength of the pharmaceutical industry’s lobbying.^48^ The IRA passed by only one vote and a simple Congressional majority could repeal its drug pricing provisions or reduce the size of the ceiling price discounts from the average market drug price.^49^ To reduce these risks, CMS will prefer not to publish an MFP without a manufacturer agreement.

The manufacturer also has an interest in making some compromises to reach an agreement. If the manufacturer and CMS do not agree on an MFP, then CMS is poised to publish an MFP that is no higher than its initial offer, which will almost certainly be lower than the statutory ceiling price. The manufacturer would then have to accept that price, incur an unsustainable tax liability, or terminate all sales to Medicare and Medicaid. To maintain its contracts and ensure the published MFP is higher than CMS’s initial proposal, the manufacturer will likely work with CMS to achieve an agreement.

Both parties will prefer an agreement to the uncertainty of a MFP published without an agreement, yet it remains unclear how they will achieve this and how much lower the MFP will be than the statutory ceiling price. In negotiating only on price, the parties are engaged in a zero-sum game, where one party’s loss is the other party’s gain. In this case, any price increase for the manufacturer implies a corresponding loss for Medicare, and vice-versa. One way to transform the negotiation into a positive-sum game that produces net gains for both parties is to include more than one item to negotiate, in this case, items in addition to the MFP. That way, each side can engage in trades and grant the other something that it wants in return for receiving something that it seeks. The party making the greater concessions on the price can receive something in return.

The IRA and its regulations do not preclude negotiating on matters in addition to price. As such, CMS and/or the manufacturer could incorporate other terms into the agreement. Due to confidentiality, we may never know what deals they make or informal understandings they have but cannot put in writing. However, negotiations are likely to involve discussions beyond the six listed fairness factors.

What items might be added to negotiation in order to reach an agreement?^50^ One possibility come to mind. CMS could ensure price confidentiality. Currently, manufacturers sell drugs at various prices, within and outside the US, providing discounts based on the purchaser’s ability to pay, their bargaining leverage, statutory restrictions, and market competition. Price confidentiality makes it harder to estimate what others pay and thereby enhances the manufacturer’s ability to engage in price discrimination.^51^

While the IRA mandates that CMS publish the MFP, it does not prohibit Medicare from paying a lower price for the drug. Therefore, in return for CMS accepting a higher MFP than it initially proposed, manufacturers could offer CMS a confidential discount, essentially agreeing to sell to CMS for a lower price than the published MFP. A high published MFP plus confidential discount would disguise the net sale price and make it easier for the manufacturer to sell drugs at premium prices to other purchasers in both the US and international markets. CMS might find this arrangement attractive if it resulted in a manufacturer acceptance of the MFP as well as a lower purchase price than it could have otherwise secured.

Manufacturers frequently employ this strategy in European markets. For example, France’s medicine pricing committee (CEPS) negotiates with each manufacturer the official maximum purchase price and simultaneously secures its agreement to pay confidential rebates lowering the net price.^52^ In the United Kingdom, manufacturers maintain list prices and employ “commercial arrangements” so that the net price is no more than deemed cost-effective by the National Institute for Health Care Excellence.^53^ Investigate Europe reports that Eli Lilly recently sought to have German legislation revised to allow confidential rebates and in return for such legislation pledged to invest in manufacturing in Germany.^54^

## Assessing Medicare Drug Price Negotiation in Light of the Discounts Obtained and Negotiation by PBMs and European Nations

Due to the confidential nature of negotiations, we lack information on what trades and concessions are made to reach an MFP. However, it would be possible to partially assess the effects of the negotiation process if we compare the published MFP to certain other prices. (We would still not know if the net price was lower than the MFP.) In August 2024, CMS published MFPs for the 10 drugs selected for negotiations completed in 2024.^55^ To highlight the savings achieved, CMS compared the published MFP to each drug’s list price, which virtually no insurer pays, but did not compare the MFPs to the prices paid by Medicaid, the VA, or to the average prices paid by Medicare drug plans in the year before the negotiation. Nor did CMS compare the MFP to the IRA statutory ceiling price for each drug (a specified discount from average market price), which lowers prices even if the manufacturer and CMS did not negotiate any further discount. Comparing the statutory ceiling price, which should be public information, to the MFP would reveal the extent to which the negotiations lowered the price from the maximum amount that the IRA allows Medicare to pay.

The prices paid by Medicaid, the VA, the statutory ceiling price and the average price paid by Medicare drug plans in the previous year were information that CMS and the manufacturer had on hand or could obtain. Publishing and comparing these prices to the MFP would better reveal the extent to which the Medicare negotiations reduced prices than a comparison to list prices. CMS’s omission in not publishing this information suggests that manufacturers and/or CMS prefer to keep as much comparative price information as possible confidential.

Two recent articles have reviewed the published maximum fair price for the first 10 drugs and estimated their relation to earlier net prices and maximum prices in other nations.

Rome et al.^56^ estimated that the Medicare MFP was probably set by the statutory ceiling price for three of the drugs. They estimated that the MFP of the other drugs were below the statutory limit and were lower than estimated Medicare net price negotiated by Part D drug plans in the year prior to the negotiation. They also found that the Medicare MFP was higher than an index of the maximum price paid by other nations although the price was similar to the index for two drugs.

Wouters et. al. found that 3 of the 10 drugs had an MFP that matched the ceiling price set by statute.^57^ Six of the other 10 drugs had an MFP below the ceiling price indicating that negotiation resulted in a price saving. The Medicare MFP was higher than the maximum prices in Australia, Canada, France, Germany, Switzerland, and the UK, with the ratio of the mean price in these nations to the Medicare MFP ranging from 2 to 4.3. There was one exception. Insulin had had its price cut substantially earlier, and so there was a 1 to 1 ratio to maximum prices in the other nations.

Neither the Rome or Wouters articles compared the Medicare MFP to Medicaid’s maximum federal price (or to estimates of Medicaid prices in states, which typically discount from the federal price) nor to the prices paid by the VA, although both Medicaid and VA prices are natural benchmarks to assess the Medicare price.

A reading of this preliminary evidence suggests three points. First, the largest savings have come from the IRA requiring at least a set discount from the average market price, a mechanism that has also worked for Medicaid. Therefore, it might be easier to achieve greater savings in the future through legislation that requires a steeper discount than by employing negotiation. Second, the US still appears to pay much more than other nations’ maximum price even before those nations receive confidential discounts. We do not know this for sure because we don’t know what undisclosed discounts CMS might have obtained that are lower than the MFP. Third, Medicare could likely obtain greater savings if it employed HTA as do other nations or if it was able to pay a price indexed to the maximum official price or the net prices paid by European nations, Canada and Australia.

As policymakers evaluate the IRA, they are also likely to compare Medicare drug price negotiation to drug price negotiation by PBMs and by European nations.

Perhaps the most important difference from negotiation by PBMs is the consequence for failing to reach an agreement. If a manufacturer does not accept CMS’s published maximum fair price it must terminate sales of all of its products to Medicare and Medicaid. In contrast, manufacturers that do not reach an agreement with a PBM typically only forgo the revenue from the sale of a single drug for an insurer that controls a much smaller market.

The scope and process of negotiation also differs. CMS only has authority to negotiate the price of a few selected designated products while PBMs can negotiate with manufacturers over the price of several or all of its products. Unlike PBMs, CMS lacks the authority to restrict patient use of drugs through high copayment or required prior authorization, and so cannot use these restrictions as leverage to secure discounts. In addition, both CMS and PBMs can refuse to purchase certain drugs if the price is too high. However, under the IRA, CMS is not permitted to purchase the selected drugs unless they receive the minimum statutory discount, while there is no minimum discount that PBMs must receive. Furthermore, CMS’s negotiation must conform to a legally regulated negotiation process while PBM negotiation does not.

**Figure 3.**
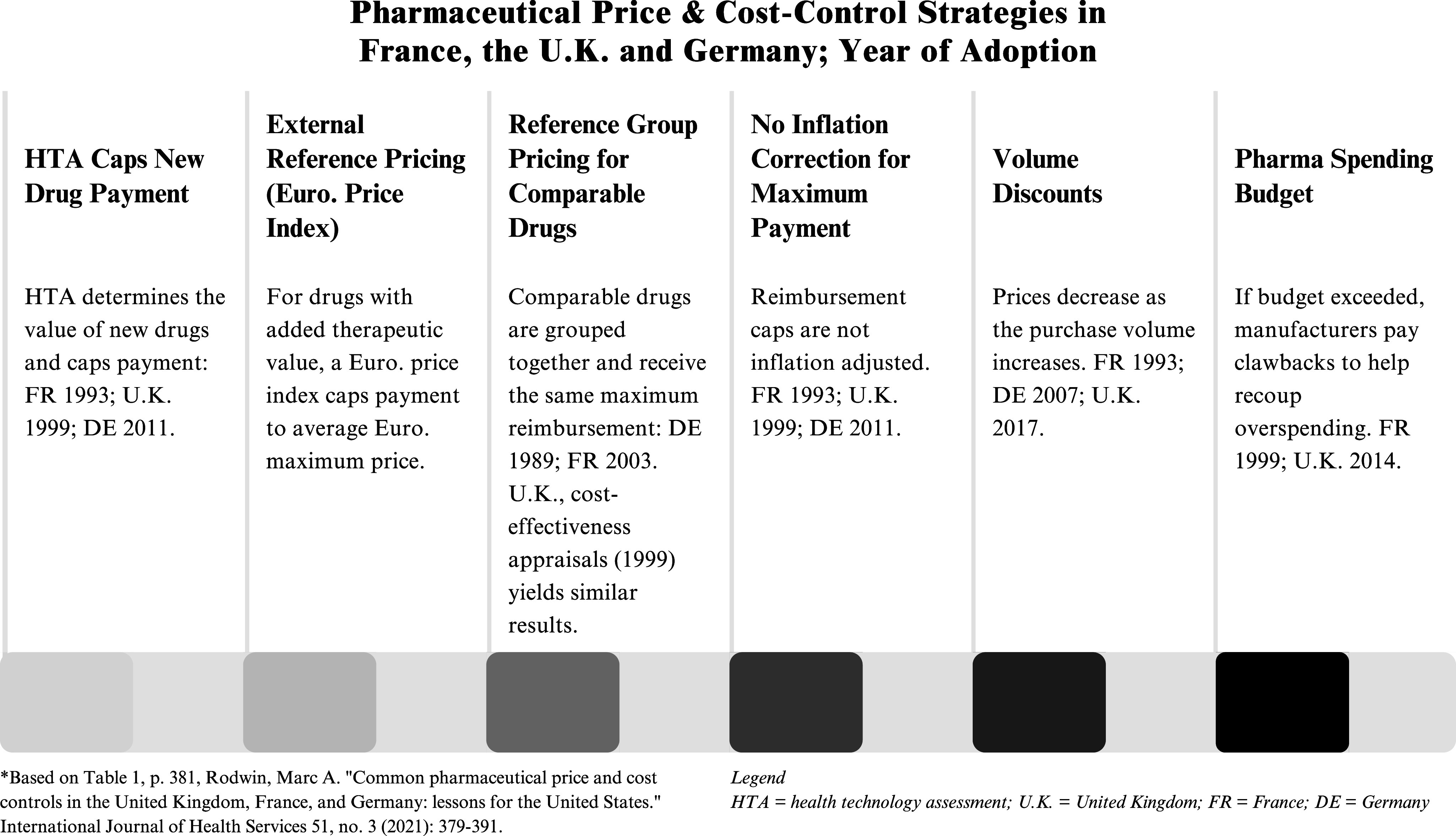
Pharmaceutical Price & Cost-Control Strategies in France, the U.K. and German; Year of Adoption.

France, Germany, the UK, and many other European nations also negotiate a maximum purchase price for drugs. There are important differences among these national systems, but they share some common elements that contrast with the negotiation process established by the IRA (see Figure 3).^58^

First, in France and the UK negotiated prices or alternative caps on purchase prices apply to all new products with the price taking effect from the first day the drug is covered (with certain exceptions in France), while in Germany the negotiated price takes effect one year after product launch. In contrast, the IRA negotiation rules apply nine to thirteen years after a drug is marketed and only for a limited number of drugs.

Second, in Europe, these negotiated maximum purchase prices apply to all purchasers. In the UK, it applies to all purchases covered by the National Health Service. In France and Germany, it applies to all the insurers that cover drugs. In contrast, the IRA rules on price negotiation apply only to Medicare.

Third, in France and Germany, price negotiation only occurs for new drugs that an independent HTA body has found offer greater therapeutic benefit than existing products. If a new drug lacks added therapeutic benefit, and many do, in place of negotiating a price, the manufacturer is paid the same price as the older comparable product. In contrast, Medicare drug price negotiation is not restricted to drugs with added therapeutic benefit.

Fourth, in France, Germany and the UK, HTA assessment helps determine the negotiated price. In the UK, cost effectiveness analysis establishes the ceiling price. In France and Germany, the HTA informs negotiators as to the drug’s added therapeutic benefit, which becomes a key element in negotiations. No IRA statutory rules or regulations direct CMS to consider HTA when negotiating the MFP.

Fifth, in France and Germany, and many other European nations, negotiators for purchasers look to the official maximum purchase price set by other European nations to cap or inform their decision on their own nation’s purchase price. The Medicare negotiation does not require that there be any US price parity with other nations, nor does it direct CMS negotiators to consider prices paid by insurers in other nations.

Sixth, some European nations, such as France and the UK, employ other tools to control pharmaceutical spending and incorporate these into any contracts negotiated, while the US lacks these other pharma spending controls. Negotiated prices are thus only one part of a strategy to control pharmaceutical spending. Both France and the UK have a national pharmaceutical spending cap set by Parliament, such that if national spending exceeds the cap, manufacturers pay rebates that reduce their sales revenue and in effect lower the sale price retroactively. UK policy allows for further reducing payments or prioritizing/controlling access to drugs unless there are additional price concessions if the cost of the drug is high enough to have a significant budget impact. France receives clawback payments if the manufacturer sales exceed those projected and agreed to in the negotiated contract. France, Germany, and the UK do not adjust maximum reimbursement prices for inflation, and they also typically obtain discounts as the volume purchased increases. In addition, for older drugs, France and Germany group these drugs together and set a single maximum reimbursement price for all drugs in the group.^59^

These differences highlight four points. First, the effect of negotiated pricing is shaped by the context in which it occurs, namely bargaining power, alternatives to a negotiated agreement, and the legal rules governing negotiation. Second, European price negotiation in continental nations follows and supplements controls on pricing derived by employing HTA. Third, negotiating prices is only one of several variables that affect prices and pharmaceutical spending, including global budgets for pharmaceutical spending and the official maximum prices in reference-price countries. Fourth, negotiated pricing in the US would have greater impact if it started at the time of product launch or soon thereafter and if it also applied to drugs outside of Medicare. American public policies will better control pharmaceutical spending if they combine negotiated pricing with other measures designed to rationalize pharmaceutical pricing and spending.

## The Relation of Medicare Negotiated Drug Prices to Private Market Prices

In summary, because US federal policy does not restrict pharmaceutical launch prices, manufacturers have set drug prices in the US higher than in other countries. Medicare Part D has historically paid particularly high prices because legislation mandates coverage of certain drugs, regardless of price, and prohibits the federal government from negotiating prices. The Inflation Reduction Act will lower the prices Medicare Part D drug plans pay for a designated number of drugs, with the number increasing over time. It sets a ceiling on the Medicare price, which is a 25 percent to 60 percent discount from average market price, with the amount increasing the longer the drug has been on the market, yet the discounts only start 9 years after marketing. It also creates a mandatory negotiation process that aims to enable Medicare to purchase drugs below the price ceiling. The new discounts ensure that Medicare receives preferential pricing compared to average private market purchasers in the US. Nevertheless, US private market branded drug prices remain the highest in the world.

Furthermore, there is no regulatory process to control the manufacturer’s launch price and Medicare only negotiates prices starting 9 to 12 years after the manufacturer receives marketing authorization. France, the UK, and Germany negotiate maximum prices at product launch and do not allow price increases thereafter. US negotiated discounts are unlikely to result in Medicare paying more than other nations pay, although this is an empirical issue that cannot be definitively answered ahead of time. If the US wants to ensure that it pays no more than European nations pay, it will need to have a means to compare its negotiated prices to the net prices paid by European nations and rules that preclude the US paying more than designated European nations.

Because the IRA drug price provisions only apply to Medicare, they do not cap the amounts that manufacturers can charge private insurers. Accordingly, without any regulation in the private market it is possible for manufacturers outside of federal programs to raise prices. Manufacturers are likely to try to increase private market prices if they can because they seek to maximize revenue, and doing so will help them make up for discounts they must grant to Medicare. If the published MFP does not reveal Medicare’s net sale price, manufacturers will have an easier time charging private insurers more than they charge Medicare. We lack data that would reveal real net prices; however, it seems likely that, unless the US employs other cost control policies, such as health technology assessment or international reference pricing that cap prices paid, it is doubtful that Medicare drug pricing will achieve parity with other comparable nations.
